# Displacement of the Cartilages of the Knee-Joint

**Published:** 1854-02

**Authors:** Edwin R. Maxson

**Affiliations:** Adams’ Centre, Jefferson county, N. Y.


ART. IV.—*Displacement of the Cartilages of the Knee-joint.* By EDWIN R. MAXSON, M. D., Adams' Centre, Jefferson county, N. Y.

The little I can find written on this subject, together with the length of time the difficulty had continued, at the time of relief, is my apology for offering the particulars of the following case for publication:

During the month of April, 1853, a Mrs. S., aged about 35 years, while standing, and resting mainly on one foot, turned the body suddenly to empty a pail of water, which she held in her hand. The motion twisted the knee-joint of the limb on which she stood, which snapped sharply, and she suddenly fell to the floor. She suffered severe pain in the joint, but after pressing and rubbing it for a time the pain grew less. Thinking the joint had been displaced, and that she had reduced it by her manipulations, she called no surgical aid at the time, thinking it would get well. She went about on crutches, but could not use the limb. It was bent a little at the knee, she swinging it has she walked. She had no power to raise the foot without grasping the limb with the hand. Thus she continued, from her own account, for about three months, with little pain or swelling of the joints. During this time she had applied two or three blisters, and used iodine ointment for a while, by medical advice.

As it grew no better, I was called, July 1st, about three months after the injury. I found her walking, as described above, by the aid of crutches without touching that foot to the floor.

On a close examination I discovered a slight prominence under the lower angle of the patella, between the inner condyle of the femur and the head of the tibia. This, with some swelling and slight tenderness about the joint, together with the history of the case, led me to the conclusion that the difficulty consisted in a displacement of the semilunar interarticular cartilages of the joint: a case of which I remembered to have read in Dorsey's Surgery. I accordingly followed out Dorsey's directions as follows: I had her seated on a board, elevated about three feet from the floor, and then, seating myself on a stool in front, and straightening the limb forward, I made counter extension with one hand above the knee, and with the other hand below the knee, I made extension; and then with both hands firmly grasped as above described, I flexed the limb, carrying the foot suddenly back under the board on which she was seated. This was repeated twice, with very little pain, when she got up and walked about the room for the first time in three months, without crutches, and with only a slight limp. There was a freeness of motion in the joint. She could raise the foot like the other, and step in at the door. The only application which I directed was a weak infusion of opium, which was continued for a few weeks. She continued to improve so that she was able to do all her work for a large family of workmen, in two months, and now, Jan. 1st, is nearly or quite well.

ADAMS' CENTRE, N. Y., Jan. 1, 1854.

